# Investigating the Inflammatory Link Between Vitamin D and Hidradenitis Suppurativa: A Systematic Review and Causal Inference Analysis

**DOI:** 10.3390/ijms27062895

**Published:** 2026-03-23

**Authors:** Jasmine Spiteri, Laura Grech, Dillon Mintoff, Nikolai P. Pace

**Affiliations:** 1 Centre for Molecular Medicine & Biobanking, University of Malta, MSD 2080 Msida, Malta; jasmine.spiteri@um.edu.mt (J.S.); laura.grech@um.edu.mt (L.G.); 2 Department of Applied Biomedical Sciences, Faculty of Health Sciences, University of Malta, MSD 2080 Msida, Malta; 3 Department of Pathology, Faculty of Medicine & Surgery, University of Malta, MSD 2080 Msida, Malta; dillon.mintoff@gov.mt; 4 Department of Dermatology, Mater Dei Hospital, MSD 2090 Msida, Malta; 5 Department of Anatomy, Faculty of Medicine & Surgery, University of Malta, MSD 2080 Msida, Malta

**Keywords:** hidradenitis suppurativa, vitamin D, Mendelian randomization, immune modulation, inflammatory marker

## Abstract

An inverse correlation between serum vitamin D levels and hidradenitis suppurativa (HS) severity is frequently reported, yet the causal nature and direction of this association remain unresolved. A systematic review was conducted following PRISMA guidelines, identifying 12 relevant studies. A two-sample Mendelian randomization (MR) analysis using the inverse-variance weighted (IVW) method was subsequently performed using genetic instruments for vitamin D from the UK Biobank (*n* = 417,580) and HS summary statistics from FinnGen (*n* = 1420). The systematic review confirmed a high prevalence of vitamin D deficiency (<20 ng mL^−1^) among HS patients (weighted mean 17.90 ng mL^−1^) and identified inverse correlations between vitamin D levels and disease severity, active lesions, and C-reactive protein (CRP), while supplementation improved clinical outcomes. A null MR estimate consistent with the absence of a detectable average linear causal effect of lifelong genetically predicted 25(OH)D levels on HS risk in the analyzed population was observed. Sensitivity analyses yielded consistent null results with no significant horizontal pleiotropy. The results suggest that hypovitaminosis D is likely a marker of the systemic inflammatory state rather than a direct causative factor. The observed clinical benefits of vitamin D supplementation warrant further interventional studies to define its potential therapeutic role.

## 1. Introduction

Vitamin D is a secosteroid prohormone involved in numerous biological processes [1,2]. Following UV-dependent synthesis from 7-dehydrocholesterol in keratinocytes or uptake from dietary sources, vitamin D undergoes sequential hydroxylation (first at C25 in the liver yielding 25-hydroxyvitamin D (25(OH)D) and subsequently (but not exclusively) in the kidney to form its active metabolite, 1α,25-dihydroxyvitamin D (1,25(OH)_2_D)) [3,4,5]. The latter circulates to target tissues where it binds to vitamin D receptor (VDR)—a ligand-dependent transcription factor of the nuclear receptor superfamily. Upon ligand binding, VDR heterodimerizes with retinoid X receptor (RXR) isotypes and recruits coactivators and co-repressors to regulate the transcription of Vitamin D-response genes [2,4].

Beyond its well-established role as a key regulator of calcium and phosphate homeostasis, vitamin D exerts pleiotropic effects across a wide range of biological processes, having complex immunoregulatory, cell growth and differentiation, and cardiovascular functions. The endocrine effects of the 1,25(OH)_2_D-VDR interaction in different cells, include lipid and phosphate absorption in intestinal enterocytes, cell cycle regulation, and regulation of immune responses. Furthermore, the conversion to active 1,25(OH)_2_D can occur extra-renally in epithelial and immune cells, enabling paracrine and autocrine signaling [1,2,4].

Vitamin D signaling serves as a regulator of both innate and adaptive immunity. Its immunomodulatory effects include enhancing the innate antibacterial response via the induction of the anti-microbial peptide cathelicidin in monocytes and macrophages. Simultaneously, it promotes a more tolerogenic immune state by steering dendritic cell differentiation towards a phenotype that favors anti-inflammatory interleukin (IL) 10 secretion, while directly suppressing the production of pro-inflammatory cytokines like IL-17 and interferon (IFN) γ in T cells. These mechanisms enhance antimicrobial defense and help maintain immune tolerance, thereby reducing the risk of autoimmunity [1,4]. The clinical relevance of this immunoregulatory role is evident in several autoimmune and chronic inflammatory conditions, including familial Mediterranean fever (FMF) [6,7,8,9], rheumatoid arthritis (RA), axial spondyloarthritis [10,11,12,13,14], and inflammatory bowel disease (IBD) [15,16,17]. In these diseases, hypovitaminosis D is not only more prevalent than in healthy controls but also frequently correlates with increased disease severity and elevated levels of proinflammatory cytokines.

Beyond its systemic immunomodulatory effects, vitamin D signaling is a regulator of cutaneous homeostasis. In the skin, the 1,25(OH)_2_D-VDR complex suppresses keratinocyte hyperproliferation and promotes their normal differentiation and the formation of the protective cornified envelope. Furthermore, it regulates hair follicle cycling through its interaction with the hairless co-repressor [4,18]. Disruption of these processes—epidermal barrier integrity, cell growth, and follicular function—provides a direct pathophysiological link between impaired vitamin D signaling and the development of chronic inflammatory skin conditions, such as psoriasis, atopic dermatitis (AD), acne vulgaris (AV), and hidradenitis suppurativa (HS) [5]. Evidence of this link is provided via meta-analytical studies that consistently report lower vitamin D levels in patients with psoriasis [19,20,21], AD [22,23,24] and AV [25,26,27] across different ages, which are negatively correlated with disease severity.

Hidradenitis suppurativa (HS) is a chronic inflammatory disease of the pilosebaceous unit that primarily affects apocrine gland-bearing regions, particularly the axillary, gluteal, and genital areas. It manifests with recurrent painful nodules that may progress into abscesses, sinus tracts, and scarring [28,29]. The etiology of HS, although incompletely understood, involves dysregulated immune responses, disruption of epidermal barrier function, cutaneous dysbiosis, and metabolic alterations as a result of the interaction between genetic and environmental factors [30].

Emerging epidemiological and clinical evidence supports a link between vitamin D deficiency and HS presentation and course. Multiple studies report an inverse association between hypovitaminosis D and HS disease severity [29,30,31,32,33,34,35]. Hypovitaminosis D has been associated with the presence of active lesions, increased levels of C-reactive protein (CRP), the co-occurrence of Crohn’s disease, and non-familial HS [29,30,32,34]. These relationships appear to be clinically relevant as continuous supplementation with vitamin D (ranging from 25,000 to 100,000 IU monthly according to the patients’ insufficiency/deficiency level) was shown to significantly improve the response to standard topical/systemic therapies as well as decrease the number of active nodules [29,31]. Nevertheless, the observational nature of these studies leaves the causal relationship between vitamin D and HS unresolved. It remains unknown whether hypovitaminosis D contributes to HS pathogenesis or if the inflammatory burden of HS leads to hypovitaminosis D.

Against this background, the present study aimed to systematically investigate the role of vitamin D in HS by: (1) reviewing and synthesizing published reports of serum vitamin D levels across different HS phenotypes and their association with demographic and clinical variables; (2) performing a two-sample Mendelian randomization (MR) analysis to determine the causal direction of the observed association; and (3) discussing the potential pathophysiological pathways linking vitamin D to HS by integrating evidence from other autoinflammatory conditions.

## 2. Materials and Methods

**Figure 1:**
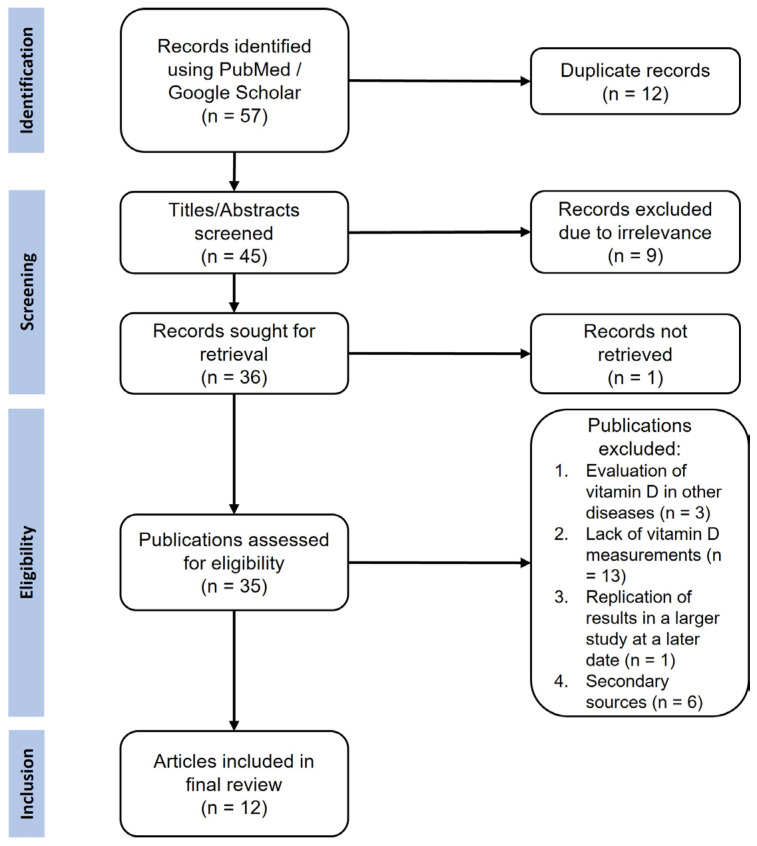
Flow diagram showing the identification of studies through database search. A total of 57 records were identified through database searching. After the removal of duplicates and screening of titles and abstracts, 35 full-text publications were assessed for eligibility. Twelve studies met the inclusion criteria and were included in the qualitative synthesis. The primary reasons for exclusion at the full-text stage were a lack of vitamin D measurements and the publication type being a secondary source (e.g., review article).

The study selection process followed the Preferred Reporting Items for Systematic Reviews and Meta-Analyses (PRISMA) guidelines, as detailed in Figure 1.

### 2.1. Study Selection and Inclusion Criteria

An electronic literature search was conducted to identify English-language studies investigating the relationship between vitamin D and HS. This search was conducted in the PubMed/MEDLINE and Google Scholar databases. The following combination of free text keywords and Medical Subject Headings terms was used for this search: “hidradenitis suppurativa”, “acne inversa”, “Verneuil’s disease”, “vitamin D”, and “25OHD”, together with the Boolean terms “AND” and “OR”. Original articles published between January 2014 and August 2025 that directly investigated the role of vitamin D in HS patients were considered eligible for inclusion. Studies that were not identified via the database search were selected through citation review and handsearching based on relevance.

### 2.2. Exclusion Criteria

Studies were excluded if they: (1) reported vitamin D levels in relation to conditions other than HS, (2) reported only observed effects of vitamin D supplementation or diet alterations in HS patients without vitamin D measurement, and (3) were conference proceedings, reviews, comments or editorial letters.

### 2.3. Data Extraction

Titles and abstracts were screened against the inclusion and exclusion criteria. Any discrepancies were resolved by consensus of the investigators. The following information was extracted from the selected articles: (1) study design, (2) measured metabolite of vitamin D and assay methodology, (3) HS phenotype, (4) patient demographic and clinical data, (5) any treatment or supplementation taken, (6) control group size (where relevant), and (7) main outcomes of the study specifically related to vitamin D.

Methodological quality of the included studies was appraised using the Mixed Methods Appraisal Tool (MMAT, version 2018), with study design-specific criteria applied to quantitative non-randomized and quantitative descriptive studies [36]. Rather than assigning an overall numeric score, we used the MMAT to identify recurrent sources of potential bias and methodological limitations relevant to interpretation of the evidence base.

### 2.4. Data Analysis

Study and patient characteristics were summarized descriptively using tables and plots. Categorical variables (sex, smoking status, and disease severity) are presented as percentages. For quantitative variables (age and BMI), a weighted mean ± weighted standard deviation was calculated, using the sample size of each individual study as the weight. This approach was applied to provide a more accurate aggregate estimate, as it assigns greater influence to studies with larger sample sizes, which are presumed to offer more precise parameter estimates.

To enable cross-study comparability, serum vitamin D levels reported in nmol L^−1^ were converted to ng mL^−1^ using the conversion factor of 2.5 (i.e., ng mL^−1^ = nmol L^−1^ ÷ 2.5). For studies reporting mean vitamin D levels with a standard deviation, the 95% confidence interval $\left(95\%\;CI=\bar{X}\pm Z\times \frac{s}{\surd n}\right)$ was calculated and used as the error bars in the forest plot to visually represent the precision of each study’s estimate Vitamin D deficiency/insufficiency categories were extracted as reported in the original studies, because threshold definitions varied (for example, <10, <20, and <30 ng/mL). Additionally, study-level reporting was insufficient for consistent reclassification thus no post hoc standardisation of categorical prevalence estimates was performed. Cross-study summaries of categorical vitamin D status were treated as descriptive and interpreted with caution [31]. A quantitative meta-analysis was not performed because serum vitamin D outcomes were reported using heterogeneous summary measures (for example, means with standard deviations or confidence intervals, and medians with ranges or interquartile ranges), and the parameters required to derive comparable effect estimates and variances were inconsistently available. In addition, individual participant data were unavailable and, in some studies, the measured vitamin D metabolite and/or assay methodology was not clearly specified, precluding reliable pooling across studies.

### 2.5. Mendelian Randomization Analysis

The two-sample MR analysis was performed using summary-level data from GWAS of European ancestry subjects. Genetic instruments for serum vitamin D levels (the exposure) were obtained from the UK Biobank (dataset ID: ebi-a-GCST90000617; *n* = 417,580) [37]. Summary statistics for hidradenitis suppurativa (the outcome) were sourced from the FinnGen Biobank (dataset ID: finn-b-L12_HIDRADENITISSUP; *n* = 1420) [38]. The analysis was performed via the MR analysis two-sample Mendelian randomization R (version 4.5.1) shiny application (MRanalysis version 2.0.0), which uses the *TwoSampleMR* and *MRPRESSO* packages [39].

SNPs associated with vitamin D levels at genome-wide significance (*p* < 5 × 10^−8^) were selected as instrumental variables (IVs). To ensure genetic independence, linkage disequilibrium (LD) clumping was performed using a strict threshold (r^2^ < 0.001) within a 10,000 kb window. SNPs with an F-statistic greater than 10 were retained to ensure strong instrument strength and mitigate potential weak instrument bias.

The primary causal estimate was derived using the IVW method. To assess the robustness of this result, we performed sensitivity analyses using the MR-Egger, weighted median, and weighted mode methods. We evaluated the validity of the model assumptions by: (1) calculating Cochran’s Q statistic to test for heterogeneity among the instrumental variable estimates; (2) applying the MR-Egger intercept test and the MR-PRESSO global test (with its leave-one-out analysis) to detect and adjust for potential horizontal pleiotropy. Variant-gene mapping of the SNPs selected as instrumental variables was carried out using snpXplorer and presented as a Circos plot [40].

## 3. Results

### 3.1. Study Characteristics

**Figure 2:**
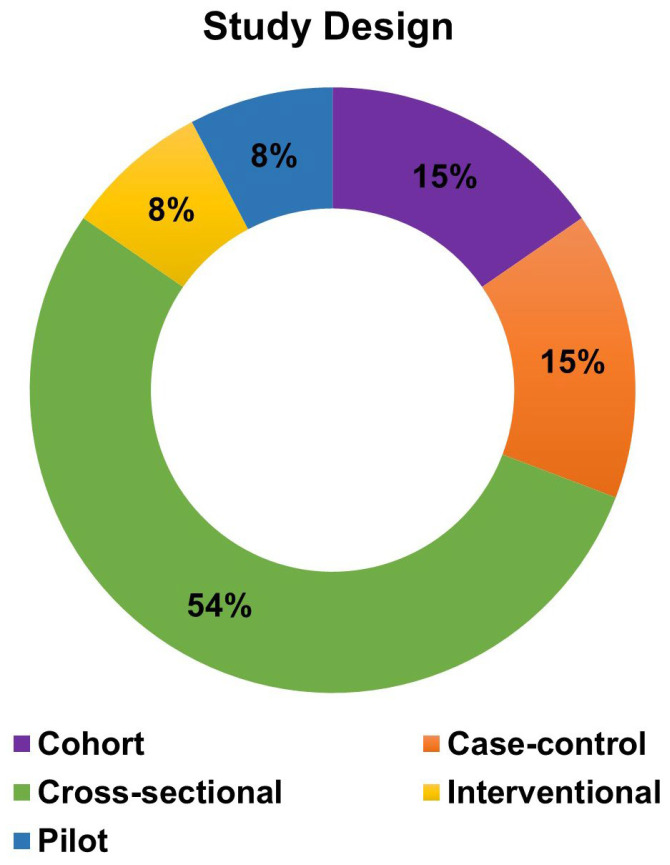
Research designs applied in the 12 selected studies, with a predominance of cross-sectional studies.

The literature search identified 35 studies investigating the role of vitamin D in HS. Following the exclusion of studies involving other skin diseases (*n* = 3), descriptive studies investigating the role of diet and vitamin D supplementation without measurements in patients (*n* = 13), superseded studies that were later replicated in larger cohorts (*n* = 1), and review articles (*n* = 6), 12 primary studies were included for analysis (Table S1) [29,30,31,32,33,34,35,41,42,43,44,45]. The number of HS patients per study ranged from 5 to 250 (*N_total_* = 956), with the majority of studies being cross-sectional and including patients with varying clinical phenotypes (Figure 2). Four studies have investigated the role of vitamin D in specific HS subpopulations, namely HS patients who have undergone bariatric surgery [41], patients with syndromic HS (Pyoderma gangrenosum, Acne, Suppurative Hidradenitis/PASH and Pyogenic Arthritis, Acne, Pyoderma gangrenosum, Suppurative Hidradenitis/PAPASH syndromes) [42], patients refractory to topical or systemic HS therapies [31], and patients diagnosed with severe HS (according to the Hurley stage and International Hidradenitis Suppurativa Severity Score System/IHS4) who are candidates for biologic therapy [43].

Among the studies that specified the methodology (*n* = 8), serum vitamin D was exclusively measured as 25-hydroxyvitamin D (25(OH)D). Electrochemiluminescence was the most common assay (*n* = 6), followed by chemiluminescence and enzyme-linked immunosorbent assay/ELISA (each *n* = 1). The remaining four studies, which were retrospective chart reviews or genomic analyses, did not specify the analyte measured and the assay used.

Demographic and clinical data were inconsistently reported across studies, with noticeable underreporting of ethnicity, the age of HS onset, disease duration, family history of HS, and the anatomical sites involved. Across the included cohorts, HS patients were predominantly adult females (mean age: 36.9 ± 4.8 years), active smokers, and with body mass indices in the obese range (mean body mass index/BMI ≥ 30 kg m^−2^) (Table 1). Most patients had moderate-severe disease (64%—combined value based on Hurley stage/IHS4/Hidradenitis Suppurativa Physician Global Assessment (HS-PGA) scoring systems), likely reflecting sampling in secondary care settings. The majority were receiving standard topical and systemic therapies for HS but had not received vitamin D supplementation for at least 6 months prior to assessment.

Information about control groups was limited. Most studies reported the inclusion of sex- and age-matched controls while only two studies accounted for smoking status and BMI. In the cohort study by Garcovich et al. (2019), HS patients without a history of bariatric surgery and exhibiting typical disease manifestations were selected as controls (according to the classification systems by Canoui-Poitrine et al. (2013) and van der Zee et al. (2015) [41,46,47]).

Structured appraisal using the Mixed Methods Appraisal Tool (MMAT) indicated that most included studies met the majority of design-specific quality criteria, particularly regarding the appropriateness of vitamin D-related measurements and the use of suitable statistical analyses for the study design (Table S2 Part I,II). Potential selection bias (single-center, retrospective, severe HS cohorts), variable control of confounding (BMI, smoking, adiposity/metabolic factors, and comorbidities), and the predominance of cross-sectional designs which limit temporal and causal inference, were identified.

### 3.2. Serum Vitamin D Level and Correlation Studies

**Figure 3:**
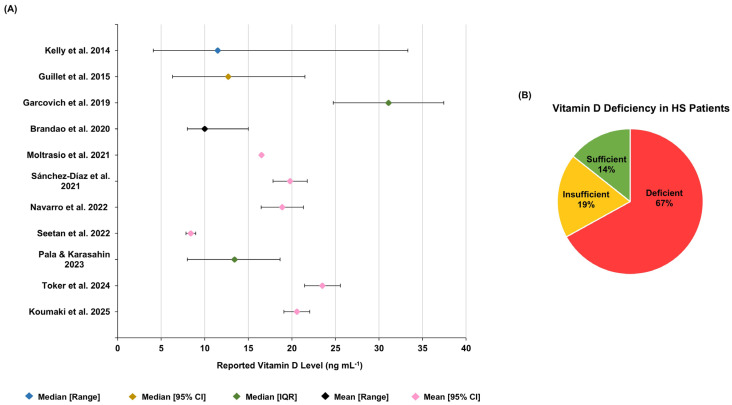
(**A**) Forest plot showing the mean or median serum vitamin D levels reported across studies (except for Fabbrocini et al. (2021) [31]). The 95% confidence interval for the mean 25(OH)D level measured by Moltrasio et al. (2021) [32] was too small (16.47–16.55) to be visualized on the plot. (**B**) Distribution of patients according to vitamin D levels as reported in the individual studies, showing a predominance of vitamin D deficient HS patients [29,30,32,33,34,35,41,42,43,44,45].

A total of 11 studies reported mean or median serum vitamin D levels in patients with HS. Across these studies, the reported central tendency values were descriptively aggregated, yielding an overall weighted mean of 17.90 ± 20.34 ng mL^−1^. This summary should be interpreted cautiously in light of substantial between-study heterogeneity in reporting format (mean versus median), dispersion metrics, and study design. Overall, serum vitamin D levels were consistently low across HS cohorts, and multiple studies reported a high frequency of vitamin D deficiency (Figure 3A,B).

Categorical vitamin D status was defined using non-uniform thresholds, with variable criteria for deficiency/sufficiency across studies. For this reason, category counts and proportions were retained according to each study’s original cut-off definitions and presented as a descriptive summary rather than directly comparable prevalence estimates to preserve fidelity to the source data and avoid introducing additional bias through post hoc reclassification. Despite these discrepancies, in the study by Koumaki et al. (2025), (where a sufficiency threshold of 20 ng mL^−1^ was applied resulting in a greater proportion of patients being classified as sufficient relative to studies using higher thresholds), the mean vitamin D level in their HS cohort remained significantly lower than in controls, consistent with the findings of four other studies [29,30,34,44,45]. The genetic landscape of syndromic HS, particularly in PASH and PAPASH phenotypes, further implicates vitamin D metabolism as a key disrupted pathway. Brandao et al. (2020) report a whole-exome sequencing study of PASH and PAPASH patients [42]. They show that approximately 45% of genes involved in vitamin D metabolism carry deleterious variants, which may significantly impair pathway functionality [42]. These genetic findings are corroborated by the consistent finding of low serum vitamin D levels, suggesting that dysregulated vitamin D signaling may also contribute to the shared pathophysiology of syndromic HS. These observations position vitamin D not merely as a nutritional correlate but as a component of a dysregulated metabolic and immunomodulatory axis in these severe, autoinflammatory forms of the disease.

Furthermore, a reduction in the number of nodules and improved response to HS therapeutics following 6 months of vitamin D supplementation, has been reported in intervention studies [29,31]. Despite the significant decrease in vitamin D levels in patients that discontinued supplementation after month 3, a statistically significant negative correlation between the number of lesions and vitamin D was observed at month 6. Additionally, the number of flare-ups was also noted to decrease after the supplementation period, highlighting the clinical relevance of vitamin D [29].

Analysis of phenotypic correlation yielded conflicting results, with some studies reporting a significant negative correlation between vitamin D levels and HS severity [30,31,32,33,34,43], and others noting no significant association between the 2 variables [29,44,45]. The presence of active lesions irrespective of anatomical site was also correlated with lower vitamin D levels [30]. Other variables, including CRP, smoking status, and BMI have also been negatively correlated with the difference in vitamin D levels in HS patients as well as the patients’ sex [32,45], but the results were not replicated by other studies [34,35]. Additionally, bariatric surgery did not influence vitamin D levels or the frequency of hypovitaminosis D in HS patients [41].

### 3.3. MR Analysis

**Figure 4:**
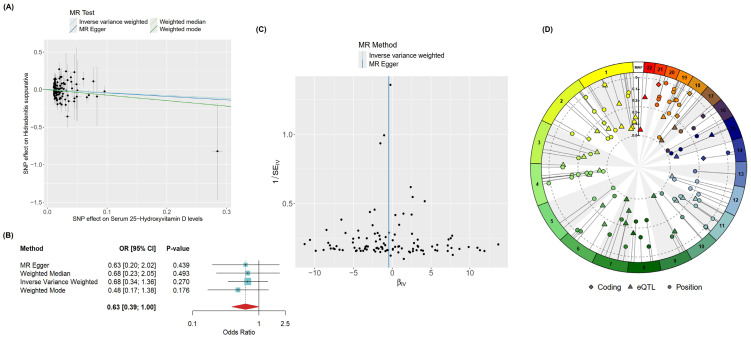
(**A**) Scatter plot and (**B**) corresponding forest plot showing no statistically significant association between vitamin D and hidradenitis suppurativa, consistent across all methods. The slightly negative sloping regression lines suggest a possible inverse relationship between vitamin D and HS, albeit unconfirmed. (**C**) Funnel plot showing an overall symmetrical distribution of effect sizes for the individual instrumental variables (IVs); these points concentrate toward the bottom of the funnel, i.e., less precise estimates which are more susceptible to pleiotropy. (**D**) Circos plot summarizing the gene mapping results of the IVs, highlighting the relatively lower number of variants in protein-coding regions and wide distribution of IVs among the chromosomes.

Given the observational nature of the findings identified in the systematic review, we employed a two-sample Mendelian randomization (MR) analysis to assess causality. This method leverages genetically predisposed variation in 25(OH)D levels to infer causal relationships, largely free from reverse causation and confounding. A total of 106 instrumental variables (IVs) were included in the MR analysis. Inverse-variance weighted (IVW) analysis does not support a linear causal effect of genetically predicted 25(OH)D on HS (*β* = −0.390, *SE* = 0.35, *p* = 0.270). Concordant results were obtained using other MR methods, as shown in Figure 4A,B. This does not exclude the possibility of non-linear, threshold-dependent, context-specific, or mediated effects that were not evaluated in the present study.

Sensitivity analyses were conducted to assess the robustness and validity of the MR findings. Cochran’s Q statistic indicated no significant heterogeneity among the variant-specific estimates (*Q_IVW_* = 126.45, *degrees of freedom* = 105, *p_IVW_* = 0.078), supporting the consistency of the genetic instruments. Furthermore, tests for horizontal pleiotropy were non-significant. Both the MR-PRESSO global test (*p* = 0.076) and the MR-Egger intercept test (*p* = 0.880) found no strong statistical evidence of pleiotropic bias. It is noteworthy, however, that the funnel plot (Figure 4C) showed an asymmetry concentrated among weaker genetic instruments, a pattern that can sometimes indicate undetected pleiotropy; nevertheless, the formal statistical tests did not confirm this.

The genetic architecture of the instrumental variables was further characterized through variant-to-gene mapping (Figure 4D). This analysis revealed that a relatively small proportion of the vitamin D-associated single nucleotide polymorphisms (SNPs) were linked to protein-coding genes, a pattern that typifies findings from genome-wide association studies (GWAS), where many trait-associated variants reside in non-coding regions such as introns or intergenic regulatory elements. This underscores the requirement for fine mapping of GWAS-identified loci to distinguish causal regulatory signals and thereby elucidate the specific biological pathways involved.

The instrumental variables demonstrated a wide genomic distribution across multiple chromosomes. This dispersion is methodologically favorable, as it reduces the likelihood of linkage disequilibrium between variants and supports the key MR assumption of genetic independence, thereby strengthening the validity of our causal inference.

An assessment of reverse causality, i.e., whether HS itself influences vitamin D levels, was not feasible. This analysis was precluded by the relatively limited sample size of the HS GWAS and the consequent lack of sufficiently strong genetic instruments to robustly test this reverse direction.

## 4. Discussion

In this study, we reconcile the well-established observational association between low vitamin D levels and HS, with MR findings that do not support a detectable linear causal effect of genetically predicted 25(OH)D levels on disease risk. Importantly, this inference is specific to the causal model tested, namely the average linear effect of lifelong genetically influenced 25(OH)D variation in a predominantly European-ancestry context, and should not be interpreted as excluding all possible causal involvement of vitamin D in HS. Rather, the findings suggest that, within this analytical framework, hypovitaminosis D may function more often as a correlate of disease phenotype, comorbidity burden, and/or shared determinants (including confounding or mediating factors), rather than as a primary etiological driver. Additionally, the estimated effect may not be directly transferable to non-European populations, where differences in allele frequencies, linkage disequilibrium structure, vitamin D distributions, and HS risk determinants could alter both instrument performance and causal estimates. Similar discrepancies between observational and genetic associations have been reported in cardiovascular disorders and inflammatory bowel disease, where the observational signal has likewise been interpreted cautiously in light of confounding, reverse causation, mediation, or non-linear/context-dependent effects [48,49,50,51].

Despite inconsistencies among MR analyses, several studies have reported inverse associations between vitamin D levels and the risk of cardiometabolic and autoimmune diseases, including hypertension, coronary heart disease, type 2 diabetes, obesity, multiple sclerosis, psoriasis, and systemic lupus erythematosus [51,52,53,54,55,56,57,58]. Several of these metabolic conditions, particularly obesity, have been linked to the development of HS, that in turn increase the risk of CVDs and metabolic syndrome in HS patients [59,60,61]. Hence, the MR studies between vitamin D and these disorders suggest a role for vitamin D in HS pathophysiology. Moreover, studies leveraging GWAS data have identified variants related to human leukocyte antigen class II molecules, which are invariably associated with autoimmune conditions and therefore imply that HS may also have an autoimmune etiology [62,63]. Thus, the involvement of vitamin D in HS merits further discussion.

### 4.1. Patient Characteristics Associated with Vitamin D

Across the reviewed studies, most HS patients were females, overweight and smokers—characteristics of the typical HS phenotype that also tend to associate with increased risk of hypovitaminosis D.

The inverse relationship between BMI or adiposity and vitamin D has been reported in several observational and MR studies [41,64,65,66,67,68]. Although individuals with a higher body surface area theoretically have a greater potential for UV-mediated 7-dehydrocholesterol photosynthesis, individuals with obesity tend to be less exposed to the sun due to the reduced outdoor activity and the use of clothing habits that cover more skin [65,66]. This tendency may be accentuated in individuals with HS, who may limit sun exposure due to visible lesions.

Apart from UVB exposure, other physiological factors influencing vitamin D levels in individuals with obesity include: (1) the increased sequestration of the lipid-soluble vitamin D in adipose tissue, (2) lower production of 25-hydroxylase due to *CYP2R1* downregulation and thus reduced formation of 25(OH)D, (3) enhanced hydroxylation of 25(OH)D in response to intact parathyroid hormone (iPTH) stimulation, and (4) VDR and vitamin D binding protein (DBP) gene polymorphisms [64,65,66,68,69,70]. The inverse causal relationship, i.e., vitamin D as a determinant of obesity could not be confirmed, mainly due to its role in the inhibition of adipogenesis and reduced accumulation of triglycerides, adipocyte apoptosis via activation of Ca^2+^-dependent signaling, regulation of lipogenesis and lipolysis, and reduction in insulin resistance [66,69,70,71,72].

Obesity and vitamin D deficiency also tend to be more prevalent in females. The significantly higher lipid levels in women have been attributed to the inhibition of HMG-CoA reductase activity by estrogen [68]. Coupled with the increased concentration of DBP and/or expression of VDR as well as the upregulated hepatic hydroxylation of vitamin D, this results in the reduced availability of biologically active vitamin [68,73,74,75,76]. Nonetheless, the results vary between studies and associations between vitamin D and testosterone have also been described, suggesting that other factors (ethnicity, diet, and health awareness) may be responsible for the discrepancy [77,78,79,80,81].

Hypovitaminosis D is also prominent in both active and passive smokers, showing decreased levels of 25(OH)D and its active metabolite 1,25(OH)_2_D. It has been suggested that the inhibition of parathyroid hormone (PTH) by smoking leads to vitamin D deficiency due to nicotine-induced hypoparathyroidism [82,83]. More research is required into the influence of smoking on vitamin D but several processes have been implicated, including: (1) impaired 1-α-hydroxylation in the kidneys, (2) increased activity of liver enzymes, (3) direct alteration of vitamin D catabolism, or (4) damage or delayed skin healing due to enhanced expression of cyclooxygenase 2 and inducible nitric oxide synthase [82,84,85,86].

Taken together, these findings suggest that vitamin D deficiency in patients with HS may largely reflect shared lifestyle with physiological characteristics rather than being disease-specific. While variation in sun exposure, BMI and smoking may exacerbate these differences, HS itself may not be a primary determinant of hypovitaminosis D.

### 4.2. Plausible Mechanistic Pathways Linking Vitamin D and Hidradenitis Suppurativa

While direct mechanistic evidence linking vitamin D to HS remains limited, existing immunologic and epithelial biology supports several plausible hypotheses. In particular, vitamin D may influence HS-relevant processes involving IL-1β/T_H_17 signaling, innate immune sensing (including NOD2-related pathways), and follicular differentiation networks. We next review potential mechanistic hypotheses linking vitamin D status to HS-relevant immune and epithelial processes.

Although the influence of sex or smoking status on HS risk remains uncertain, obesity is a well-established predisposing factor. Excess weight exacerbates HS not only through mechanical friction but also through meta-inflammation driven by adipocyte hypertrophy and macrophage infiltration, resulting in increased IL-1β, IL-6, IL-8, tumor necrosis factor (TNF) α levels via toll-like receptor (TLR) mediated NF-κB activation [69,70,73,87,88,89,90]. Both 25(OH)D and 1,25(OH)_2_D have been shown to attenuate these inflammatory pathways, reducing TNF-α and IL-6 expression in adipose tissue [73,87]. This suggests that vitamin D supplementation might hinder the inflammatory response in individuals with HS and obesity and that inflammatory mediators may be involved in the observed relationship between vitamin D and other HS phenotypes.

Parallels are provided from studies investigating the role of vitamin D in other inflammatory diseases such as rheumatological and dermatological conditions which share pathogenic pathways with HS. In psoriatic plaques and AV inflammatory lesions, treatment with vitamin D or its analogues resulted in reduced T_H_17-mediated immunity in keratinocytes via inhibition of T-cell recruitment and NF-κB signaling [91,92,93]. Similar observations were also made in patients with RA, where low levels of vitamin D are associated with increased formation of reactive oxygen species (ROS) that ultimately activates NF-κB and elevates the T_H_17-related cytokine IL-21 [14]. Despite the lack of a dominant T_H_ axis in HS, IL-17 signaling is a major contributor to the development of the inflammatory cascade, through immune cell infiltration of both uninvolved and lesional skin and the upregulation of antimicrobial peptides (AMPs) like psoriasin [94,95,96,97,98]. The development of T_H_17 cells and hence release of IL-17 cytokine is enhanced by the increased levels of the upstream regulator IL-1β [98,99]. This has also been targeted by topical vitamin D treatment in acne vulgaris, suggesting that vitamin D treatment could mitigate the pro-inflammatory milieu resulting from the IL-1β-T_H_17 cell cytokine axis in HS [100]. Other pro-inflammatory markers such as IFN-γ and TNF-α, which are also upregulated in HS patients and associated with keratinocyte hyperproliferation, have also been downregulated in AD and IBD most likely through VDR signaling [97,101,102,103,104,105]. Additionally, an increase in AMPs, such as psoriasin (S100A7) and cathelicidin (LL-37) in keratinocytes and sebocytes was also noted in psoriasis and AV [91,92,93]. The keratinocytic induction of cathelicidin in response to vitamin D as opposed to its release from neutrophils in HS patients, would prevent microbial infections of lesional skin without aggravating inflammation [91,106]. Vitamin D may thus influence inflammatory pathways relevant to HS through effects on innate and adaptive immune responses, although direct HS-specific mechanistic evidence remains limited.

Conversely, vitamin D positively regulates Notch signaling in the intestinal barrier, preserving the integrity of tight junctions [107]. Differentially expressed genes related to the Notch pathway that are regulated by VDR have also been identified in HS lesions, but enhanced Notch signaling has been associated with keratinocyte hyperproliferation and innate immunity activation which would lead to inflammation [108,109]. Similarly, 1,25(OH)_2_D stimulates the pathogen-recognition receptor NOD2 in IBD, triggering inflammation and innate immunity dysregulation. NOD2 has also been identified in the HS inflammasome, particularly in syndromic HS, and thus treatment with vitamin D could exacerbate inflammation [110,111,112]. Plausibly, NOD2-linked innate immune dysregulation represents a potential pathway for vitamin D-related inflammatory variation in HS, but this remains speculative and hypothesis-generating.

The metabolite 1,25(OH)_2_D has also been associated with hair follicle cycle regulation. This involves shortening of the anagen phase potentially through the Wnt/β-catenin pathway to promote hair regeneration and enhancing proliferation and migration of follicular cells [113]. These processes are VDR-dependent and likely involve downregulation of the HIF-1α and NLRP3, both being overexpressed in HS patients [113,114,115]. Since HIF-1α promotes glycolytic metabolism, angiogenesis and IL-1β/IL-17 [114], and NLRP3 mediates IL-1β and IL-18 maturation, vitamin D-mediated suppression of these factors may mitigate inflammation and follicular occlusion, the hallmarks of HS pathogenesis [98,113,114,115]. Nonetheless, these mechanistic pathways should be interpreted as hypothesis-generating models that may help contextualize the observed clinical associations between vitamin D status and HS, rather than as directly causal mechanisms.

The observed clinical improvements in HS (reduction in the number of nodules and frequency of flare-ups, and enhanced response to therapy following vitamin D supplementation) collectively support a modulatory rather than causal role for Vitamin D in HS [29,31]. However, further interventional trials are warranted to determine the usefulness, dosage and formulation of vitamin D as an adjuvant therapeutic in the management of HS.

### 4.3. Methodological Variations in Serum Vitamin D Measurements

The measurement of the same metabolite (25(OH)D) in at least 8 of the reviewed studies using similar analysis methods, yielded different mean values of vitamin D even in patient groups of similar ethnicities. Aside from methodological limitations (variability in sample size and patient demographic/clinical characteristics), other confounding factors may be implicated. These include factors involving UVB exposure such as melanin pigmentation or religious customs, changes in 7-dehydrocholesterol levels with age, gene polymorphisms, liver/kidney disease, and interfering medication [29,30,45,116]. Numerous articles have reviewed the issues related to vitamin D measurements, not only those pertaining to physiological aspects, but also inter-laboratory and inter-assay variations [116,117,118].

From a laboratory perspective, the accurate quantification of vitamin D status is hampered by significant methodological heterogeneity. Commonly employed immunoassays are susceptible to variable cross-reactivity with different vitamin D metabolites and epimers which have differing affinity constants to binding proteins. Additionally, these assays are biased by sample matrix interference, particularly in the presence of lipids. Despite the increased specificity of chromatographic methods, their application is limited to research purposes while clinical settings favor automated and rapid immunoassays [116,118,119,120]. The lack of agreement on the utilized assay influences the interpretation of serum 25(OH)D levels across studies, particularly in cases where cut-off values are specified according to the kit used or a specific condition (usually musculoskeletal) being studied, without taking into consideration the different roles of vitamin D and general health [118,121,122]. Furthermore, the sole reliance on total 25(OH)D as a biomarker is potentially limiting, as it does not reflect the bioavailable fraction or the influence of other relevant metabolites whose concentrations may be differentially detected by various assays. [118,121,123]. The measurement of active free metabolites may thus inform the definition of the physiological ranges of vitamin D, provided methods are developed to accurately measure these unstable molecules. The absence of measurements of additional laboratory parameters, such as serum PTH, in most clinical cohorts presents an additional limitation, as the PTH-25(OH)D relationship is a key dynamic regulator of calcium homeostasis and its omission prevents a more integrated physiological assessment of functional vitamin D status [124]. This data would help inform more holistic guidelines that take into consideration the broader role and benefits of vitamin D beyond bone health, as well as variations in its metabolism in different individuals, to ensure adequate dosaging [125,126].

These limitations can greatly impact the outcomes of the studies included in this review, as shown through their conflicting correlation analyses. Furthermore, the influence of these confounding factors indicates that the use of genetic instruments in a linear MR analysis may not be representative of the true biological association between serum 25(OH)D and HS. The non-linear relationship between vitamin D and other biomarkers suggests that applying a stratified, non-linear MR model or the application of mediation analysis could better explain the role of vitamin D in HS. Other limitations include: (1) the GWAS data is based on European participants and therefore the results cannot be extrapolated to other populations; (2) residual bias cannot be excluded since the exact biological function of the IVs is not known; (3) canalization or compensatory developmental processes may dampen the effect of genetically dysregulated 25(OH)D, favoring a null result. Additional limitations merit consideration. Primarily, no formal meta-analysis of serum vitamin D levels was performed due to substantial methodological and reporting heterogeneity across studies, incomplete variance data, and inconsistent specification of the vitamin D analyte/assay in some reports. These factors limit validity and interpretability of a pooled estimate. Furthermore, the reported heterogeneity in vitamin D threshold definitions across studies introduces potential classification bias and constrains direct comparison of deficiency/insufficiency prevalence. In the absence of sufficiently granular published data, post hoc harmonization of categories was not consistently possible. The observational evidence base is also subject to methodological limitations identified on MMAT appraisal including potential selection bias, inconsistent adjustment for important confounders (notably BMI and smoking), and predominantly cross-sectional study designs, all of which may influence the observed association between vitamin D status and HS.

## 5. Conclusions

Overall, the low vitamin D levels in HS patients identified by previous studies cannot be confirmed to cause HS by linear MR analysis; reverse causality also cannot be ascertained. Nonetheless, the modulation of immune and inflammatory responses, specifically the IL-1β-T_H_17 cell cytokine axis, as well as the regulation of the hair cycle and keratinocyte proliferation, highlight possible mechanistic avenues through which vitamin D may improve HS symptoms. Further understanding of HS pathophysiology, the interplay between vitamin D supplements and administered HS therapeutics as well as other confounders is necessary, prior to inclusion of vitamin D in a treatment regimen.

## Figures and Tables

**Table 1:** Summary of the most reported patient characteristics across the 12 studies. The arithmetic mean (sex, smoking status, HS severity) and weighted mean (age, BMI) were determined from the reported values.

Variable	HS Patients
**Sex** (*n* = 956, %)	
*Female*	56
*Male*	44
**Age** (years)	36.9 ± 4.8
**BMI** (kg m^−2^)	30.03 ± 3.57
**Smoking Status, active** (*n* = 668, %)	65
**HS Severity** (*n* = 916, %)	
*Mild*	36
*Moderate-Severe*	64

## Data Availability

The GWAS data used in this study are made available by the UK Biobank and the FinnGen research group, with reference numbers ebi-a-GCST90000617 (vitamin D) and finn-b-L12_HIDRADENITISSUP (HS), respectively. The data was retrieved directly through the online MR analysis application: https://www.mranalysis.cn/ (accessed on 21 July 2025).

## Supplementary Materials

The following supporting information can be downloaded at: https://www.mdpi.com/article/10.3390/ijms27062895/s1.

## Abbreviations

The following abbreviations are used in this manuscript:

25(OH)D	25-hydroxyvitamin D
AD	Atopic Dermatitis
AMP	Antimicrobial Peptide
AV	Acne Vulgaris
BMI	Body Mass Index
CRP	C reactive Protein
CVD	Cardiovascular Disorder
DBP	Vitamin D Binding Protein
ELISA	Enzyme-Linked Immunosorbent Assay
FMF	Familial Mediterranean Fever
GWAS	Genome-Wide Association Study
HS	Hidradenitis Suppurativa
HS-PGA	Hidradenitis Suppurativa Physician Global Assessment
IBD	Inflammatory Bowel Disease
IFN	Interferon
IHS4	International Hidradenitis Suppurativa Severity Score System
IL	Interleukin
iPTH	Intact Parathyroid Hormone
IV	Instrumental Variable
IVW	Inverse-Variance Weighted
LD	Linkage Disequilibrium
MMAT	Mixed Methods Appraisal Tool
MR	Mendelian Randomization
PAPASH	Pyogenic Arthritis, Acne, Pyoderma gangrenosum, Suppurative Hidradenitis
PASH	Pyoderma gangrenosum, Acne, Suppurative Hidradenitis
PRISMA	Preferred Reporting Items for Systematic Reviews and Meta-Analyses
PTH	Parathyroid Hormone
RA	Rheumatoid Arthritis
ROS	Reactive Oxygen Species
RXR	Retinoid X Receptor
SNP	Single Nucleotide Polymorphism
TLR	Toll-Like Receptor
TNF	Tumor Necrosis Factor
VDR	Vitamin D Receptor

## References

[1] Verstuyf A., Carmeliet G., Bouillon R., Mathieu C. (2010). Vitamin D: A Pleiotropic Hormone. Kidney Int..

[2] Rochel N. (2022). Vitamin D and Its Receptor from a Structural Perspective. Nutrients.

[3] DeLuca H.F., Zierold C. (2009). Mechanisms and Functions of Vitamin D. Nutr. Rev..

[4] Haussler M.R., Whitfield G.K., Kaneko I., Haussler C.A., Hsieh D., Hsieh J.-C., Jurutka P.W. (2013). Molecular Mechanisms of Vitamin D Action. Calcif. Tissue Int..

[5] Bukvić Mokos Z., Tomić Krsnik L., Harak K., Marojević Tomić D., Tešanović Perković D., Vukojević M. (2025). Vitamin D in the Prevention and Treatment of Inflammatory Skin Diseases. Int. J. Mol. Sci..

[6] Erten Ş., Altunoğlu A., Ceylan G.G., Maraş Y., Koca C., Yüksel A. (2012). Low Plasma Vitamin D Levels in Patients with Familial Mediterranean Fever. Rheumatol. Int..

[7] Kisacik B., Kaya S.U., Pehlivan Y., Tasliyurt T., Sayarlioglu M., Onat A.M. (2013). Decreased Vitamin D Levels in Patients with Familial Mediterranean Fever. Rheumatol. Int..

[8] Turhan T., Doğan H.O., Boğdaycioğlu N., Eyerci N., Omma A., Sari İ., Yeşilyurt A., Karaaslan Y. (2018). Vitamin D Status, Serum Lipid Concentrations, and Vitamin D Receptor (VDR) Gene Polymorphisms in Familial Mediterranean Fever. Bosn. J. Basic Med. Sci..

[9] Kozan M., Ozan Z.T., Demir V., Ede H. (2019). The Relation of Novel Cardiovascular Risk Parameters in Patients with Familial Mediterranean Fever. JRSM Cardiovasc. Dis..

[10] Rossini M., Maddali Bongi S., La Montagna G., Minisola G., Malavolta N., Bernini L., Cacace E., Sinigaglia L., Di Munno O., Adami S. (2010). Vitamin D Deficiency in Rheumatoid Arthritis: Prevalence, Determinants and Associations with Disease Activity and Disability. Arthritis Res. Ther..

[11] Zhao S., Thong D., Duffield S., Goodson N. (2017). Vitamin D Deficiency in Axial Spondyloarthritis Is Associated with Higher Disease Activity. Arch. Rheumatol..

[12] Meena N., Singh Chawla S.P., Garg R., Batta A., Kaur S. (2018). Assessment of Vitamin D in Rheumatoid Arthritis and Its Correlation with Disease Activity. J. Nat. Sci. Biol. Med..

[13] Lin J., Liu J., Davies M.L., Chen W. (2016). Serum Vitamin D Level and Rheumatoid Arthritis Disease Activity: Review and Meta-Analysis. PLoS ONE.

[14] Shufang M., Xiaojiao H., Yinhong K. (2024). Pro-Inflammatory Cytokine IL-21 Correlates with the Reactive Oxygen Species and 25-Hydroxy Vitamin D in Rheumatoid Arthritis Patients. Immun. Inflamm. Dis..

[15] Konjeti V.R., Vaziri H., Soriano M. (2011). Role of Vitamin D in Inflammatory Bowel Disease Flare: P-88. Inflamm. Bowel Dis..

[16] Del Pinto R., Pietropaoli D., Chandar A.K., Ferri C., Cominelli F. (2015). Association Between Inflammatory Bowel Disease and Vitamin D Deficiency: A Systematic Review and Meta-Analysis. Inflamm. Bowel Dis..

[17] Yang C.-T., Yen H.-H., Su P.-Y., Chen Y.-Y., Huang S.-P. (2024). High Prevalence of Vitamin D Deficiency in Taiwanese Patients with Inflammatory Bowel Disease. Sci. Rep..

[18] Bikle D.D. (2011). Vitamin D Metabolism and Function in the Skin. Mol. Cell Endocrinol..

[19] Pitukweerakul S., Thavaraputta S., Prachuapthunyachart S., Karnchanasorn R. (2019). Hypovitaminosis D Is Associated with Psoriasis: A Systematic Review and Meta-Analysis. Kans. J. Med..

[20] Formisano E., Proietti E., Borgarelli C., Pisciotta L. (2023). Psoriasis and Vitamin D: A Systematic Review and Meta-Analysis. Nutrients.

[21] Moosazadeh M., Damiani G., Khademloo M., Kheradmand M., Nabinezhad-Male F., Hessami A. (2023). Comparing Vitamin D Level Between Patients with Psoriasis and Healthy Individuals: A Systematic Review and Meta-Analysis. J. Evid. Based Complement. Altern. Med..

[22] Kim M.J., Kim S.-N., Lee Y.W., Choe Y.B., Ahn K.J. (2016). Vitamin D Status and Efficacy of Vitamin D Supplementation in Atopic Dermatitis: A Systematic Review and Meta-Analysis. Nutrients.

[23] Hattangdi-Haridas S.R., Lanham-New S.A., Wong W.H.S., Ho M.H.K., Darling A.L. (2019). Vitamin D Deficiency and Effects of Vitamin D Supplementation on Disease Severity in Patients with Atopic Dermatitis: A Systematic Review and Meta-Analysis in Adults and Children. Nutrients.

[24] Ng J.C., Yew Y.W. (2022). Effect of Vitamin D Serum Levels and Supplementation on Atopic Dermatitis: A Systematic Review and Meta-Analysis. Am. J. Clin. Dermatol..

[25] Acharya P., Mathur M. (2020). Vitamin D Deficiency in Patients with Acne Vulgaris: A Systematic Review and Meta-analysis. Australas. J. Dermatol..

[26] Wang M., Zhou Y., Yan Y. (2021). Vitamin D Status and Efficacy of Vitamin D Supplementation in Acne Patients: A Systematic Review and Meta-Analysis. J. Cosmet. Dermatol..

[27] Hasamoh Y., Thadanipon K., Juntongjin P. (2022). Association between Vitamin D Level and Acne, and Correlation with Disease Severity: A Meta-Analysis. Dermatology.

[28] Zouboulis C.C., Bechara F.G., Fritz K., Goebeler M., Hetzer F.H., Just E., Kirsten N., Kokolakis G., Kurzen H., Nikolakis G. (2024). S2k Guideline for the Treatment of Hidradenitis Suppurativa/Acne Inversa—Short Version. J. Dtsch. Dermatol. Ges..

[29] Guillet A., Brocard A., Bach Ngohou K., Graveline N., Leloup A.G., Ali D., Nguyen J.-M., Loirat M.J., Chevalier C., Khammari A. (2015). Verneuil’s Disease, Innate Immunity and Vitamin D: A Pilot Study. J. Eur. Acad. Dermatol. Venereol..

[30] Pala E., Karasahin O. (2023). Does Vitamin D Deficiency Exacerbate Hidradenitis Suppurativa?. Med. Sci..

[31] Fabbrocini G., Marasca C., Luciano M.A., Guarino M., Poggi S., Fontanella G., Cacciapuoti S. (2021). Vitamin D Deficiency and Hidradenitis Suppurativa: The Impact on Clinical Severity and Therapeutic Responsivity. J. Dermatol. Treat..

[32] Moltrasio C., Tricarico P.M., Genovese G., Gratton R., Marzano A.V., Crovella S. (2021). 25-Hydroxyvitamin D Serum Levels Inversely Correlate to Disease Severity and Serum C-Reactive Protein Levels in Patients with Hidradenitis Suppurativa. J. Dermatol..

[33] Toker M., Ch’en P.Y., Rangu S., Campton K.L., Cohen S.R. (2024). Vitamin D Deficiency May Be Associated with Severity of Hidradenitis Suppurativa: A Retrospective Cohort Analysis of a Racially and Ethnically Diverse Patient Population. Int. J. Dermatol..

[34] Koumaki D., Gregoriou S., Evangelou G., Rovithi E., Koumaki V., Petrou D., Solia Apokidou E., Ioannou P., Katoulis A., Papadakis M. (2025). Vitamin D Deficiency as a Predictor of Hidradenitis Suppurativa Severity. Int. J. Dermatol..

[35] Kelly G., Sweeney C.M., Fitzgerald R., O’Keane M.P., Kilbane M., Lally A., Tobin A.M., McKenna M.J., Kirby B. (2014). Vitamin D Status in Hidradenitis Suppurativa. Br. J. Dermatol..

[36] Hong Q.N., Pluye P., Fàbregues S., Bartlett G., Boardman F., Cargo M., Dagenais P., Gagnon M.-P., Griffiths F., Nicolau B. Mixed Methods Appraisal Tool Version 2018: User Guide. http://mixedmethodsappraisaltoolpublic.pbworks.com/.

[37] Revez J.A., Lin T., Qiao Z., Xue A., Holtz Y., Zhu Z., Zeng J., Wang H., Sidorenko J., Kemper K.E. (2020). Genome-Wide Association Study Identifies 143 Loci Associated with 25 Hydroxyvitamin D Concentration. Nat. Commun..

[38] Kurki M.I., Karjalainen J., Palta P., Sipilä T.P., Kristiansson K., Donner K.M., Reeve M.P., Laivuori H., Aavikko M., Kaunisto M.A. (2023). FinnGen Provides Genetic Insights from a Well-Phenotyped Isolated Population. Nature.

[39] Li K., Xing A., Cai T., Li Z. MRanalysis. https://www.mranalysis.cn/.

[40] Tesi N., van der Lee S., Hulsman M., Holstege H., Reinders M.J.T. snpXplorer. https://snpxplorer.net/.

[41] Garcovich S., De Simone C., Giovanardi G., Robustelli E., Marzano A.V., Peris K. (2019). Post-bariatric Surgery Hidradenitis Suppurativa: A New Patient Subset Associated with Malabsorption and Micronutritional Deficiencies. Clin. Exp. Dermatol..

[42] Brandao L., Moura R., Tricarico P.M., Gratton R., Genovese G., Moltrasio C., Garcovich S., Boniotto M., Crovella S., Marzano A.V. (2020). Altered Keratinization and Vitamin D Metabolism May Be Key Pathogenetic Pathways in Syndromic Hidradenitis Suppurativa: A Novel Whole Exome Sequencing Approach. J. Dermatol. Sci..

[43] Sánchez-Díaz M., Salvador-Rodríguez L., Montero-Vílchez T., Martínez-López A., Arias-Santiago S., Molina-Leyva A. (2021). Cumulative Inflammation and HbA1c Levels Correlate with Increased Intima-Media Thickness in Patients with Severe Hidradenitis Suppurativa. J. Clin. Med..

[44] Navarro I., González-López M.A., Sierra I., Olmos J.M., Blanco R., Hernández J.L. (2022). Bone Metabolism in Patients with Hidradenitis Suppurativa: A Case-Control Study. Acta Derm. Venereol..

[45] Seetan K., Eldos B., Saraireh M., Omari R., Rubbai Y., Jayyusi A., Jubran M.A. (2022). Prevalence of Low Vitamin D Levels in Patients with Hidradenitis Suppurativa in Jordan: A Comparative Cross-Sectional Study. PLoS ONE.

[46] Canoui-Poitrine F., Thuaut A.L., Revuz J.E., Viallette C., Gabison G., Poli F., Pouget F., Wolkenstein P., Bastuji-Garin S. (2013). Identification of Three Hidradenitis Suppurativa Phenotypes: Latent Class Analysis of a Cross-Sectional Study. J. Investig. Dermatol..

[47] Van Der Zee H.H., Jemec G.B.E. (2015). New Insights into the Diagnosis of Hidradenitis Suppurativa: Clinical Presentations and Phenotypes. J. Am. Acad. Dermatol..

[48] Sofianopoulou E., Kaptoge S.K., Afzal S., Jiang T., Gill D., Gundersen T.E., Bolton T.R., Allara E., Arnold M.G., Mason A.M. (2024). Estimating Dose-Response Relationships for Vitamin D with Coronary Heart Disease, Stroke, and All-Cause Mortality: Observational and Mendelian Randomisation Analyses. Lancet Diabetes Endocrinol..

[49] Lund-Nielsen J., Vedel-Krogh S., Kobylecki C.J., Brynskov J., Afzal S., Nordestgaard B.G. (2018). Vitamin D and Inflammatory Bowel Disease: Mendelian Randomization Analyses in the Copenhagen Studies and UK Biobank. J. Clin. Endocrinol. Metab..

[50] Huang T., Afzal S., Yu C., Guo Y., Bian Z., Yang L., Millwood I.Y., Walters R.G., Chen Y., Chen N. (2019). Vitamin D and Cause-Specific Vascular Disease and Mortality: A Mendelian Randomisation Study Involving 99,012 Chinese and 106,911 European Adults. BMC Med..

[51] Afzal S., Brøndum-Jacobsen P., Bojesen S.E., Nordestgaard B.G. (2014). Vitamin D Concentration, Obesity, and Risk of Diabetes: A Mendelian Randomisation Study. Lancet Diabetes Endocrinol..

[52] Vimaleswaran K.S., Cavadino A., Berry D.J., Jorde R., Dieffenbach A.K., Lu C., Alves A.C., Heerspink H.J.L., Tikkanen E., Eriksson J. (2014). Association of Vitamin D Status with Arterial Blood Pressure and Hypertension Risk: A Mendelian Randomisation Study. Lancet Diabetes Endocrinol..

[53] Zhang Z., Qiu S., Wang Z., Hu Y. (2024). Vitamin D Levels and Five Cardiovascular Diseases: A Mendelian Randomization Study. Heliyon.

[54] Lu L., Bennett D.A., Millwood I.Y., Parish S., McCarthy M.I., Mahajan A., Lin X., Bragg F., Guo Y., Holmes M.V. (2018). Association of Vitamin D with Risk of Type 2 Diabetes: A Mendelian Randomisation Study in European and Chinese Adults. PLoS Med..

[55] Huang Y.Y., Zhang W.S., Jiang C.Q., Zhu F., Jin Y.L., Cheng K.K., Lam T.H., Xu L. (2023). Mendelian Randomization on the Association of Obesity with Vitamin D: Guangzhou Biobank Cohort Study. Eur. J. Clin. Nutr..

[56] Mokry L.E., Ross S., Ahmad O.S., Forgetta V., Smith G.D., Leong A., Greenwood C.M.T., Thanassoulis G., Richards J.B. (2015). Vitamin D and Risk of Multiple Sclerosis: A Mendelian Randomization Study. PLoS Med..

[57] Zhang Y., Jing D., Zhou G., Xiao Y., Shen M., Chen X., Liu H. (2022). Evidence of a Causal Relationship Between Vitamin D Status and Risk of Psoriasis from the UK Biobank Study. Front. Nutr..

[58] Zhao S.S., Mason A., Gjekmarkaj E., Yanaoka H., Burgess S. (2023). Associations between Vitamin D and Autoimmune Diseases: Mendelian Randomization Analysis. Semin. Arthritis Rheum..

[59] Van Straalen K.R., Vanlaerhoven A.M.J.D., Ardon C.B., van der Zee H.H. (2021). Body Mass Index at the Onset of Hidradenitis Suppurativa. J. Dtsch. Dermatol. Ges..

[60] Mintoff D., Pace N. (2025). Causal Association between Body Fat Percentage and Hidradenitis Suppurativa: A Two-Sample Mendelian Randomization Study. J. Eur. Acad. Dermatol. Venereol..

[61] Miller I.M., Ellervik C., Vinding G.R., Zarchi K., Ibler K.S., Knudsen K.M., Jemec G.B.E. (2014). Association of Metabolic Syndrome and Hidradenitis Suppurativa. JAMA Dermatol..

[62] Bao A., Kollings J., Ma E., Manjunath J., D’Amiano A., Driscoll M.S., Kwatra S.G. (2025). Association of Human Leukocyte Antigen Allelic Variants with Hidradenitis Suppurativa across Fitzpatrick Skin Types: A Cross-Sectional Analysis. J. Am. Acad. Dermatol..

[63] Zierer J., Cojean C., Osinga R., Läuchli S., Wieczorek G., Roth L., Roediger B. (2025). Hidradenitis Suppurativa Is an HLA-Associated Autoimmune Disease. J. Investig. Dermatol..

[64] Vimaleswaran K.S., Berry D.J., Lu C., Tikkanen E., Pilz S., Hiraki L.T., Cooper J.D., Dastani Z., Li R., Houston D.K. (2013). Causal Relationship between Obesity and Vitamin D Status: Bi-Directional Mendelian Randomization Analysis of Multiple Cohorts. PLoS Med..

[65] Lagunova Z., Porojnicu A., Lindberg F., Hexeberg S., Moan J. (2009). The Dependency of Vitamin D Status on Body Mass Index, Gender, Age and Season. Obes. Metabol..

[66] Pereira-Santos M., Costa P.R.F., Assis A.M.O., Santos C.A.S.T., Santos D.B. (2015). Obesity and Vitamin D Deficiency: A Systematic Review and Meta-Analysis. Obes. Rev..

[67] Lespessailles E., Toumi H. (2017). Vitamin D Alteration Associated with Obesity and Bariatric Surgery. Exp. Biol. Med..

[68] Li X., Liu Y., Wang J., Chen X., Reichetzeder C., Elitok S., Krämer B.K., Doebis C., Huesker K., von Baehr V. (2024). Vitamin D Is Associated with Lipid Metabolism: A Sex- and Age-Dependent Analysis of a Large Outpatient Cohort. Nutrients.

[69] Ruiz-Ojeda F.J., Anguita-Ruiz A., Leis R., Aguilera C.M. (2018). Genetic Factors and Molecular Mechanisms of Vitamin D and Obesity Relationship. Ann. Nutr. Metab..

[70] Szymczak-Pajor I., Miazek K., Selmi A., Balcerczyk A., Śliwińska A. (2022). The Action of Vitamin D in Adipose Tissue: Is There the Link between Vitamin D Deficiency and Adipose Tissue-Related Metabolic Disorders?. Int. J. Mol. Sci..

[71] Sergeev I.N. (2020). Vitamin D Status and Vitamin D-Dependent Apoptosis in Obesity. Nutrients.

[72] Feghaly J., Johnson P., Kalhan A. (2020). Vitamin D and Obesity in Adults: A Pathophysiological and Clinical Update. Br. J. Hosp. Med..

[73] Roy P., Nadeau M., Valle M., Bellmann K., Marette A., Tchernof A., Gagnon C. (2015). Vitamin D Reduces LPS-Induced Cytokine Release in Omental Adipose Tissue of Women but Not Men. Steroids.

[74] Wierzbicka A., Oczkowicz M. (2022). Sex Differences in Vitamin D Metabolism, Serum Levels and Action. Br. J. Nutr..

[75] Muscogiuri G., Barrea L., Somma C.D., Laudisio D., Salzano C., Pugliese G., de Alteriis G., Colao A., Savastano S. (2019). Sex Differences of Vitamin D Status across BMI Classes: An Observational Prospective Cohort Study. Nutrients.

[76] Verdoia M., Schaffer A., Barbieri L., Di Giovine G., Marino P., Suryapranata H., De Luca G. (2015). Impact of Gender Difference on Vitamin D Status and Its Relationship with the Extent of Coronary Artery Disease. Nutr. Metab. Cardiovasc. Dis..

[77] Hagenau T., Vest R., Gissel T.N., Poulsen C.S., Erlandsen M., Mosekilde L., Vestergaard P. (2009). Global Vitamin D Levels in Relation to Age, Gender, Skin Pigmentation and Latitude: An Ecologic Meta-Regression Analysis. Osteoporos. Int..

[78] Smith M. (2010). Seasonal, Ethnic and Gender Variations in Serum Vitamin D3 Levels in the Local Population of Peterborough. Biosci. Horiz..

[79] Lippi G., Montagnana M., Meschi T., Borghi L. (2012). Vitamin D Concentration and Deficiency across Different Ages and Genders. Aging Clin. Exp. Res..

[80] Rafiq R., van Schoor N.M., Sohl E., Zillikens M.C., Oosterwerff M.M., Schaap L., Lips P., de Jongh R.T. (2016). Associations of Vitamin D Status and Vitamin D-Related Polymorphisms with Sex Hormones in Older Men. J. Steroid Biochem. Mol. Biol..

[81] AlQuaiz A.M., Kazi A., Fouda M., Alyousefi N. (2018). Age and Gender Differences in the Prevalence and Correlates of Vitamin D Deficiency. Arch. Osteoporos..

[82] Brot C., Rye Jørgensen N., Helmer Sørensen O. (1999). The Influence of Smoking on Vitamin D Status and Calcium Metabolism. Eur. J. Clin. Nutr..

[83] Cutillas-Marco E., Fuertes-Prosper A., Grant W.B., Morales-Suárez-Varela M. (2012). Vitamin D Deficiency in South Europe: Effect of Smoking and Aging. Photodermatol. Photoimmunol. Photomed..

[84] Supervía A., Nogués X., Enjuanes A., Vila J., Mellibovsky L., Serrano S., Aubía J., Díez-Pérez A. (2006). Effect of Smoking and Smoking Cessation on Bone Mass, Bone Remodeling, Vitamin D, PTH and Sex Hormones. J. Musculoskelet. Neuronal Interact..

[85] Nwosu B.U., Kum-Nji P. (2018). Tobacco Smoke Exposure Is an Independent Predictor of Vitamin D Deficiency in US Children. PLoS ONE.

[86] Yang L., Zhao H., Liu K., Wang Y., Liu Q., Sun T., Chen S., Ren L. (2021). Smoking Behavior and Circulating Vitamin D Levels in Adults: A Meta-Analysis. Food Sci. Nutr..

[87] Palaniswamy S., Gill D., De Silva N.M., Lowry E., Jokelainen J., Karhu T., Mutt S.J., Dehghan A., Sliz E., Chasman D.I. (2020). Could Vitamin D Reduce Obesity-Associated Inflammation? Observational and Mendelian Randomization Study. Am. J. Clin. Nutr..

[88] Sun X., Zemel M.B. (2008). Calcitriol and Calcium Regulate Cytokine Production and Adipocyte–Macrophage Cross-Talk. J. Nutr. Biochem..

[89] Boer J., Jemec G.B.E. (2021). Mechanical Forces and Hidradenitis Suppurativa. Exp. Dermatol..

[90] Mintoff D., Agius R., Benhadou F., Das A., Frew J.W., Pace N.P. (2023). Obesity and Hidradenitis Suppurativa: Targeting Meta-Inflammation for Therapeutic Gain. Clin. Exp. Dermatol..

[91] Peric M., Koglin S., Dombrowski Y., Groß K., Bradac E., Büchau A., Steinmeyer A., Zügel U., Ruzicka T., Schauber J. (2009). Vitamin D Analogs Differentially Control Antimicrobial Peptide/“Alarmin” Expression in Psoriasis. PLoS ONE.

[92] Lim S.-K., Ha J.-M., Lee Y.-H., Lee Y., Seo Y.-J., Kim C.-D., Lee J.-H., Im M. (2016). Comparison of Vitamin D Levels in Patients with and without Acne: A Case-Control Study Combined with a Randomized Controlled Trial. PLoS ONE.

[93] Papa V., Li Pomi F., Minciullo P.L., Borgia F., Gangemi S. (2025). Skin Disorders and Osteoporosis: Unraveling the Interplay Between Vitamin D, Microbiota, and Epigenetics Within the Skin–Bone Axis. Int. J. Mol. Sci..

[94] Jenei A., Dajnoki Z., Medgyesi B., Gáspár K., Béke G., Kinyó Á., Méhes G., Hendrik Z., Dinya T., Törőcsik D. (2019). Apocrine Gland–Rich Skin Has a Non-Inflammatory IL-17–Related Immune Milieu, That Turns to Inflammatory IL-17–Mediated Disease in Hidradenitis Suppurativa. J. Investig. Dermatol..

[95] Kim J., Lee J., Li X., Lee H.S., Kim K., Chaparala V., Murphy W., Zhou W., Cao J., Lowes M.A. (2023). Single-Cell Transcriptomics Suggest Distinct Upstream Drivers of IL-17A/F in Hidradenitis versus Psoriasis. J. Allergy Clin. Immunol..

[96] Ben Abdallah H., Bregnhøj A., Iversen L., Johansen C. (2023). Transcriptomic Analysis of Hidradenitis Suppurativa: A Unique Molecular Signature with Broad Immune Activation. Int. J. Mol. Sci..

[97] Navrazhina K., Garcet S., Zheng X., Hur H.B., Frew J.W., Krueger J.G. (2022). High Inflammation in Hidradenitis Suppurativa Extends to Perilesional Skin and Can Be Subdivided by Lipocalin-2 Expression. J. Allergy Clin. Immunol..

[98] Kelly G., Hughes R., McGarry T., van den Born M., Adamzik K., Fitzgerald R., Lawlor C., Tobin A.M., Sweeney C.M., Kirby B. (2015). Dysregulated Cytokine Expression in Lesional and Nonlesional Skin in Hidradenitis Suppurativa. Br. J. Dermatol..

[99] Witte-Händel E., Wolk K., Tsaousi A., Irmer M.L., Mößner R., Shomroni O., Lingner T., Witte K., Kunkel D., Salinas G. (2019). The IL-1 Pathway Is Hyperactive in Hidradenitis Suppurativa and Contributes to Skin Infiltration and Destruction. J. Investig. Dermatol..

[100] Kwaśna J., Bychowski M., Górski M., Załęska A., Kaźmierczyk I., Lenart K., Homza M., Zakrzewska N., Bednarek S., Kulicka J. (2024). The Role of Vitamin D in Acne Vulgaris: A Comprehensive Review of Recent Advances. Qual. Sport..

[101] Matusiak L., Bieniek A., Szepietowski J.C. (2009). Increased Serum Tumour Necrosis Factor-α in Hidradenitis Suppurativa Patients: Is There a Basis for Treatment with Anti-Tumour Necrosis Factor-α Agents?. Acta Derm. Venereol..

[102] De Oliveira A.S.L.E., Bloise G., Moltrasio C., Coelho A., Agrelli A., Moura R., Tricarico P.M., Jamain S., Marzano A.V., Crovella S. (2022). Transcriptome Meta-Analysis Confirms the Hidradenitis Suppurativa Pathogenic Triad: Upregulated Inflammation, Altered Epithelial Organization, and Dysregulated Metabolic Signaling. Biomolecules.

[103] Di Filippo P., Scaparrotta A., Rapino D., Cingolani A., Attanasi M., Petrosino M.I., Chuang K., Di Pillo S., Chiarelli F. (2015). Vitamin D Supplementation Modulates the Immune System and Improves Atopic Dermatitis in Children. Int. Arch. Allergy Immunol..

[104] Kellermann L., Hansen S.L., Maciag G., Granau A.M., Johansen J.V., Teves J.M., Bressan R.B., Pedersen M.T., Soendergaard C., Baattrup A.M. (2024). Influence of Vitamin D Receptor Signalling and Vitamin D on Colonic Epithelial Cell Fate Decisions in Ulcerative Colitis. J. Crohns Colitis.

[105] Heine G., Hoefer N., Franke A., Nöthling U., Schumann R.R., Hamann L., Worm M. (2013). Association of Vitamin D Receptor Gene Polymorphisms with Severe Atopic Dermatitis in Adults. Br. J. Dermatol..

[106] Thomi R., Schlapbach C., Yawalkar N., Simon D., Yerly D., Hunger R.E. (2018). Elevated Levels of the Antimicrobial Peptide LL-37 in Hidradenitis Suppurativa Are Associated with a Th1/Th17 Immune Response. Exp. Dermatol..

[107] Li Y., Guo Y., Geng C., Song S., Yang W., Li X., Wang C. (2024). Vitamin D/Vitamin D Receptor Protects Intestinal Barrier against Colitis by Positively Regulating Notch Pathway. Front. Pharmacol..

[108] Hessam S., Gambichler T., Skrygan M., Scholl L., Sand M., Meyer T., Stockfleth E., Bechara F.G. (2021). Increased Expression Profile of NCSTN, Notch and PI3K/AKT3 in Hidradenitis Suppurativa. J. Eur. Acad. Dermatol. Venereol..

[109] Gauntner T.D. (2019). Hormonal, Stem Cell and Notch Signalling as Possible Mechanisms of Disease in Hidradenitis Suppurativa: A Systems-level Transcriptomic Analysis. Br. J. Dermatol..

[110] Fletcher J., Cooper S.C., Ghosh S., Hewison M. (2019). The Role of Vitamin D in Inflammatory Bowel Disease: Mechanism to Management. Nutrients.

[111] Marzano A.V., Genovese G., Moltrasio C., Tricarico P.M., Gratton R., Piaserico S., Garcovich S., Boniotto M., Brandão L., Moura R. (2022). Whole-Exome Sequencing in 10 Unrelated Patients with Syndromic Hidradenitis Suppurativa: A Preliminary Step for a Genotype-Phenotype Correlation. Dermatology.

[112] Jfri A., Litvinov I.V., Netchiporouk E., O’Brien E. (2020). Novel Variants of MEFV and NOD2 Genes in Familial Hidradenitis Suppurativa: A Case Report. SAGE Open Med. Case Rep..

[113] Zong X., Yang S., Tang Z., Li X., Long D., Wang D. (2024). 1,25-(OH)2D3 Promotes Hair Growth by Inhibiting NLRP3/IL-1β and HIF-1α/IL-1β Signaling Pathways. J. Nutr. Biochem..

[114] Agamia N.F., Sorror O.A., Sayed N.M., Ghazala R.A., Echy S.M., Moussa D.H., Melnik B.C. (2023). Overexpression of Hypoxia-Inducible Factor-1α in Hidradenitis Suppurativa: The Link between Deviated Immunity and Metabolism. Arch. Dermatol. Res..

[115] Krajewski P.K., Szukała W., Szepietowski J.C. (2024). The NLRP3 Inflammasome Gene Is Overexpressed in Hidradenitis Suppurativa Lesions: A Preliminary Study on the Role of Pyroptosis in Disease Pathogenesis. Curr. Issues Mol. Biol..

[116] Volmer D.A., Mendes L.R.B.C., Stokes C.S. (2015). Analysis of Vitamin D Metabolic Markers by Mass Spectrometry: Current Techniques, Limitations of the “Gold Standard” Method, and Anticipated Future Directions. Mass. Spectrom. Rev..

[117] Snellman G., Melhus H., Gedeborg R., Byberg L., Berglund L., Wernroth L., Michaëlsson K. (2010). Determining Vitamin D Status: A Comparison between Commercially Available Assays. PLoS ONE.

[118] Máčová L., Bičíková M. (2021). Vitamin D: Current Challenges between the Laboratory and Clinical Practice. Nutrients.

[119] Wallace A.M., Gibson S., de la Hunty A., Lamberg-Allardt C., Ashwell M. (2010). Measurement of 25-Hydroxyvitamin D in the Clinical Laboratory: Current Procedures, Performance Characteristics and Limitations. Steroids.

[120] Altieri B., Cavalier E., Bhattoa H.P., Pérez-López F.R., López-Baena M.T., Pérez-Roncero G.R., Chedraui P., Annweiler C., Della Casa S., Zelzer S. (2020). Vitamin D Testing: Advantages and Limits of the Current Assays. Eur. J. Clin. Nutr..

[121] Sempos C.T., Heijboer A.C., Bikle D.D., Bollerslev J., Bouillon R., Brannon P.M., DeLuca H.F., Jones G., Munns C.F., Bilezikian J.P. (2018). Vitamin D Assays and the Definition of Hypovitaminosis D: Results from the First International Conference on Controversies in Vitamin D. Br. J. Clin. Pharmacol..

[122] Grimnes G., Almaas B., Eggen A.E., Emaus N., Figenschau Y., Hopstock L.A., Hutchinson M.S., Methlie P., Mihailova A., Sneve M. (2010). Effect of Smoking on the Serum Levels of 25-Hydroxyvitamin D Depends on the Assay Employed. Eur. J. Endocrinol..

[123] Bartoszewicz Z., Kondracka A., Jaźwiec R., Popow M., Dadlez M., Bednarczuk T. (2013). Can We Accurately Measure the Concentration of Clinically Relevant Vitamin D Metabolites in the Circulation? The Problems and Their Consequences. Endokrynol. Pol..

[124] Pludowski P., Takacs I., Boyanov M., Belaya Z., Diaconu C.C., Mokhort T., Zherdova N., Rasa I., Payer J., Pilz S. (2022). Clinical Practice in the Prevention, Diagnosis and Treatment of Vitamin D Deficiency: A Central and Eastern European Expert Consensus Statement. Nutrients.

[125] Demay M.B., Pittas A.G., Bikle D.D., Diab D.L., Kiely M.E., Lazaretti-Castro M., Lips P., Mitchell D.M., Murad M.H., Powers S. (2024). Vitamin D for the Prevention of Disease: An Endocrine Society Clinical Practice Guideline. J. Clin. Endocrinol. Metab..

[126] Grant W.B., Wimalawansa S.J., Pludowski P., Cheng R.Z. (2025). Vitamin D: Evidence-Based Health Benefits and Recommendations for Population Guidelines. Nutrients.

